# OphthoEvidence report: Baseline structural optical coherence tomography biomarkers as predictors of visual outcomes following full-thickness macular hole surgery: A systematic review and meta-analysis protocol

**DOI:** 10.1371/journal.pone.0359108

**Published:** 2026-09-30

**Authors:** Sahar Khademioore, João Pedro Lima, Dena Zeraatkar, Sadia Mahmood, Sara Khademioureh, Charles C. Wykoff, Peter K. Kaiser, Tien-Yin Wong, Jiayuan Cheng, Mengda Li, Varun Chaudhary

**Affiliations:** 1 Department of Health Research Methods, Evidence, and Impact, McMaster University, Hamilton, ON, Canada; 2 Thomas Kevill Advanced Vitreoretinal Imaging (AVI) Lab, St Joseph’s Healthcare, Hamilton, ON, Canada; 3 Department of Surgery, McMaster University, Hamilton, ON, Canada; 4 Department of Anesthesia, McMaster University, Hamilton, ON, Canada; 5 School of Public Health, University of Alberta, Edmonton, Alberta, Canada; 6 Retina Consultants of Texas, Houston, Texas, United States of America; 7 Cole Eye Institute, Cleveland Clinic, Cleveland, Ohio, United States of America; 8 Beijing Visual Science and Translational Eye Research Institute, School of Clinical Medicine, Beijing Tsinghua Changgung Hospital, Tsinghua Medicine, Tsinghua University, Beijing, China; 9 Singapore Eye Research Institute, Singapore National Eye Centre, Singapore, Singapore; 10 Tsinghua Medicine, Tsinghua University, Beijing, China; 11 School of Biomedical Engineering, Tsinghua Medicine, Tsinghua University, Beijing, China; University of Florida College of Medicine, UNITED STATES OF AMERICA

## Abstract

**Background:**

Full-thickness macular hole (FTMH) is a vitreoretinal condition affecting the foveal center that causes central vision loss and metamorphopsia. Despite high surgical success in achieving closure, functional visual recovery remains unpredictable. Identifying reliable optical coherence tomography (OCT) biomarkers that predict postoperative outcomes could improve patient counselling and surgical decision-making.

**Objective:**

To systematically evaluate the predictive value of baseline structural OCT biomarkers for visual acuity (VA) outcomes at 3, 6, 12, and 24 months following FTMH surgery.

**Methods:**

We will search PubMed, EMBASE, Cochrane Library, and Web of Science databases from inception for studies evaluating baseline OCT parameters as predictors of postoperative VA outcomes following FTMH surgery, with minimum follow-up of 3 months. Data will be synthesized using random-effects meta-analyses. The certainty of evidence will be assessed using the Grading of Recommendations Assessment, Development and Evaluation (GRADE) approach.

**Conclusions:**

This systematic review will synthesize current evidence on OCT biomarkers’ predictive value for FTMH surgical outcomes. The findings will clarify their clinical utility and may inform individualized prognostic counselling and surgical decision-making, including which patients are most likely to benefit from surgery and which surgical techniques may be most effective given specific OCT profiles.

## 1. Introduction

Full-thickness macular hole (FTMH) is a vitreoretinal interface condition characterized by a complete break in all neurosensory retinal layers at the foveal center [1]. The condition affects approximately 3–8 per 100,000 of the population per year, with peak incidence in the sixth and seventh decades of life, predominantly affecting female sex [2–4].

Current surgical management consists of pars plana vitrectomy with internal limiting membrane (ILM) peeling and gas tamponade, with anatomical closure rates surpassing 95% [5,6]. However, functional visual recovery varies significantly among patients, with some achieving excellent visual rehabilitation while others experience limited improvement [4]. This variability in visual outcomes creates challenges in patient counselling and management decisions.

Optical coherence tomography (OCT) has changed the diagnosis, staging, and monitoring of macular holes, providing high-resolution cross-sectional images of retinal architecture, enabling precise morphometric analysis of structural features that correlate with surgical outcomes [7].

Advances in OCT have identified many structural biomarkers with potential prognostic value for predicting visual outcomes following FTMH surgery. Traditional parameters such as minimum linear diameter, base diameter, and calculated indices, including the macular hole index and tractional hole index, have demonstrated varying degrees of predictive accuracy across multiple studies [7–11]. Recent studies have explored novel OCT biomarkers, including choroidal hypertransmission width, external limiting membrane integrity, and ellipsoid zone restoration patterns, with some showing strong correlations with visual outcomes [12–14]. Additionally, artificial intelligence and machine learning approaches using OCT-derived features have shown promising results in outcome prediction [15]. With this growing body of evidence on the potential predictive value of OCT biomarkers for visual outcomes, a comprehensive and rigorous review is essential to synthesize the current evidence, identify predictive parameters, and enable evidence-based integration into personalized treatment pathways for FTMH patients.

This systematic review and meta-analysis will evaluate the predictive accuracy of baseline structural OCT biomarkers for visual acuity (VA) at 3-, 6-, 12-, and 24-months following FTMH surgery.

## 2. Methods

### 2.1. Protocol and registration

This systematic review protocol is reported according to the Preferred Reporting Items for Systematic Reviews and Meta-Analyses Protocols (PRISMA-P) statement [16]. We register our protocol on the Open Science Framework (OSF) (http://osf.io/).

### 2.2. Eligibility criteria

This review will include randomized controlled trials (RCTs), or observational studies (cohort, case–control, or case series with more than 20 participants) that include patients with FTMH undergoing primary vitrectomy and assess the association of baseline OCT biomarkers with VA with a minimum of 3 months follow-up from surgery. Eligible pars plana vitrectomy (PPV) procedures include PPV alone or combined phacovitrectomy, regardless of baseline lens status (phakic or pseudophakic) with or without internal limiting membrane (ILM) peeling (standard peel, limited versus wide peel), ILM flap techniques (inverted-ILM flap, temporal/inverted variants, free ILM flap), and other primary graft/plug techniques (autologous neurosensory retinal transplant, autologous lens-capsule flap, human amniotic membrane plug, or autologous platelet/serum adjuncts). We will include studies reporting any tamponade agent (air; SF6; C3F8; silicone oil) and any post-operative positioning protocol (Table 1). Studies including patients any stages of FTMH will be considered for inclusion. We will consider only baseline (preoperative) structural OCT biomarkers as prognostic factors; postoperative OCT features will not be assessed. We will exclude studies of revisions and reoperations and reports not including primary data such as review articles, editorials, and commentaries. Studies focused primarily on developing, validating, or testing formal prediction equations/models, and machine learning algorithms will be excluded.

**Table 1:** Inclusion and Exclusion Criteria.

Criteria	Description
INCLUSION CRITERIA	
Study Design	• RCTs • Observational studies (including cohort studies, case-control and case series>=20 participants)
Population	• FTMH: ◦ Idiopathic or myopic ◦ Gass stage 2, 3, or 4 macular holes AND • Patients undergoing primary vitrectomy including pars plana vitrectomy alone or combined phacovitrectomy: ◦ With or without ILM peeling ◦ Any tamponade agent (e.g., air, gas, or silicone oil) ◦ Any baseline lens status (phakic or pseudophakic) ◦ Both standard and inverted ILM flap techniques ◦ Other primary graft/plug techniques (e.g., autologous neurosensory retinal transplant, autologous lens capsule flap, human amniotic membrane plug, or autologous platelet/serum adjuncts)
Prognostic factors	• Baseline OCT biomarkers measured preoperatively • Quantitative OCT parameters (e.g., hole diameter, height, base diameter) • Morphological OCT features • Any OCT-derived measurements assessed for prognostic value
Outcomes	• Final BCVA score • Change in BCVA from baseline • Macular hole closure rate • VRQoL
**EXCLUSION CRITERIA**	
Study Design	• Review articles • Reports not including primary data • Case reports with insufficient data
Population	• Lamellar macular hole, macular pseudohole, or impending macular hole only • Traumatic macular hole • Macular hole associated with retinal detachment (MHRD) or myopic traction maculopathy / foveoschisis • Secondary macular hole (e.g., age-related macular degeneration, diabetic retinopathy, retinal vein occlusion)

Abbreviations: RCT: Randomized controlled trial; FTMH: Full-thickness macular hole; ILM: Internal limiting membrane; OCT: Optical coherence tomography; BCVA: Best-corrected visual acuity; VR-QoL: Vision-related quality of life.

### 2.3. Information sources

In collaboration with a medical librarian, we will search MEDLINE, EMBASE, Cochrane Central Register of Controlled Trials (CENTRAL), and Web of Science from inception, using terms related to FTMH, OCT, prognostic factors, and vitrectomy. Additionally, we will consult clinical experts in the field to identify any potentially missing studies. We will also supplement our search with forward and backward citation searching using *citationchaser* (https://estech.shinyapps.io/citationchaser/).

### 2.4. Study selection

Two independent reviewers working in duplicate will screen titles and abstracts of search records, and subsequently the full-texts of records deemed potentially eligible at the title and abstract screening stage, using Covidence (Veritas Health Innovation, Melbourne, Australia). Reviewers will resolve disagreements through discussion or consultation with a third reviewer. A PRISMA flow diagram will document the study selection process.

### 2.5. Data collection process

Reviewers, working independently and in duplicate, will collect data using a standardized, piloted data extraction form built using Microsoft Excel (Version 16.0). Reviewers will resolve disagreements through discussion or consultation with a third reviewer. Data on study characteristics, participant demographics, imaging parameters, surgical details, outcome assessment and statistical analysis will be extracted (Table 2).

**Table 2:** Data Extraction Framework.

Category	Data Elements
Study characteristics	First author, study design, setting, country, sample size, publication year, funding source, eligibility criteria, follow-up duration.
Participant demographics	Age, sex distribution, eye laterality, prior retinal surgery, baseline visual acuity, symptom duration before surgery, comorbidities (diabetes, hypertension, myopia (axial length and refractive error)), Gass stage classification for macular hole and hole size according to International Vitreomacular Traction Study Group classification [17].
Imaging parameters	OCT parameters and their definitions, OCT measurement methods and equipment, including whether an image reading centre was used to identify the biomarkers
Surgical interventions	Type of vitrectomy, combined phacoemulsification, ILM strategy (e.g., ILM peeling, flap technique), use of grafts/plugs, dyes, tamponade agent, duration of surgery, tamponade status at the time of outcome assessment (e.g., silicone oil in situ or removed), and lens status at outcome assessment (including cataract surgery performed during follow-up).
Outcome measures and analysis	Visual acuity: visual acuity assessment methods, OCT-visual acuity associations by time point, follow-up duration and intervals, statistical methods for analysis (univariate or multivariate), and variables included in multivariate models Anatomical closure success rates

Abbreviations: OCT: Optical coherence tomography; ILM: Internal limiting membrane.

### 2.6. Data synthesis

We will stratify analyses by postoperative time point (i.e., 3, 6, 12, and 24 months) and outcome. Each timepoint will include data at or, if not reported, ± 2 months of the timepoint of interest. Meta-analysis will be performed if 2 or more studies demonstrate sufficient homogeneity in terms of population characteristics, OCT measurement methods, outcome measures, and statistical reporting. Pooled estimates for prognostic factors will be calculated using the restricted maximum likelihood (REML) random-effects models. All analyses will be performed in R (Version 4.3.1) using the *meta* and *metafor* packages [18,19]. When meta-analysis is not feasible, we will summarize findings narratively following Synthesis Without Meta-analysis (SWiM) guidance [20]. In addition, because the presence of silicone oil tamponade and changes in lens status (e.g., cataract progression or subsequent cataract extraction) at the time of outcome assessment may affect measured visual acuity, we will conduct sensitivity analyses excluding studies in which visual acuity was assessed with silicone oil in situ. Also, when available, we will extract stratified results based on lens and silicone oil status.

**Outcomes:**

We will assess the ability of baseline OCT biomarkers to predict visual outcomes following surgery defined by:

1. Final best-corrected visual acuity (BCVA) at 3, 6, 12, and 24 months postoperatively,
2. Change in BCVA from baseline at the same postoperative time points,
3. Macular hole closure rate following surgery, defined as complete apposition of the margins,
4. Vision-related quality of life (VR-QoL).

VA measurements will be accepted in any standardized format including Logarithm of the Minimum Angle of Resolution (logMAR) notation, Snellen fraction, or Early Treatment Diabetic Retinopathy Study (ETDRS) letter score. We will convert values reported in logMAR, Snellen chart scores or any other validated BCVA scale to approximate ETDRS scores using the previously established method for statistical analysis to ensure comparability across studies [21]. We will determine the approach for VRQoL score conversions, if any, after carefully assessing the number of studies using each scale and the construct measured by each instrument.

### 2.7. Subgroup analysis

Planned subgroup analyses will be conducted based on hole size categories (small holes defined as ≤ 400 micrometers versus large holes greater than 400 micrometers) [17], Gass staging (stage 2, 3 versus 4), surgical techniques (internal limiting membrane peeling versus no peeling, standard versus inverted flap techniques, PPV-alone versus combined phacovitrectomy), myopia status (myopic versus non-myopic eyes) and risk of bias (high versus low risk of bias). Subgroup analyses will be performed only when at least two studies are available within each subgroup; given that the number of studies per subgroup may be limited, subgroup findings will be interpreted cautiously. For statistically significant subgroup effects, we will use the Instrument to assess the Credibility of Effect Modification Analyses (ICEMAN) tool to assess the credibility of the subgroup analyses [22]. For subgroup effects deemed to be of moderate or high credibility, we will only present stratified results.

### 2.8. Risk of bias assessment

A pair of independent reviewers will assess the risk of bias using the Quality in Prognosis Studies (QUIPS) tool [23], and disagreements will be resolved through discussion. Risk of bias will be assessed as low, moderate or high based on the following domains: participation, attrition, prognostic factor measurement, confounding measurement and account, outcome measurement, and statistical analysis and reporting. In the confounding domain, we will rate the evidence as low risk if studies identify and appropriately adjust for baseline visual acuity, macular hole size/minimum linear diameter, hole duration, tamponade agent, surgical technique (ILM peeling/flap strategy), age and sex [6]. In the outcome measurement domain, we will additionally consider whether visual acuity was assessed with silicone oil in situ or in eyes with visually significant cataract, as these factors may systematically bias measured visual acuity.

### 2.9. Certainty of evidence

We will assess the certainty of evidence as high, moderate, low, or very low for each association using the Grading of Recommendations, Assessment, Development and Evaluation (GRADE) approach for assessment of evidence about prognostic factors [24]. We will rate certainty according to the risk of bias of the studies, imprecision, inconsistency, indirectness, and publication bias.

We will assess imprecision using a minimally contextualized approach, defining the minimal clinically important difference (MCID) as a visual acuity improvement of ≥15 ETDRS letters [25,26]. Even though there is no consensus, we will consider 10% —value used as a non-inferiority margin in trials— as MCID for macular hole closure rate [27].

Heterogeneity will be assessed using visual inspection of forest plots regarding the direction of point estimates, the overlap in confidence intervals, statistical heterogeneity (i.e., chi-square test) and the I² statistic.

Publication bias will be assessed using funnel plots and Egger's test when ≥10 studies are available; for fewer studies, we will not downgrade GRADE certainty for publication bias due to insufficient power of detection methods but will consider publication bias qualitatively in the evidence assessment by assessing the comprehensiveness of our search strategy and evidence acquisition methods [28].

## 3. Discussion

This systematic review aims to provide comprehensive evidence on the predictive value of baseline OCT biomarkers for visual outcomes following FTMH surgery at 3, 6, 12, and 24 months after surgery.

This study employs several methodological approaches to address current evidence gaps and minimize bias. First, the comprehensive search strategy across multiple electronic databases, supplemented by forward and backward citation searching, will maximize study identification and reduce the risk of missing relevant studies. Second, the implementation of duplicate screening and data extraction processes by independent reviewers will minimize selection bias and reduce the risk of data extraction errors. The use of standardized, piloted data extraction forms will ensure consistent and comprehensive data collection across studies. Third, the application of GRADE methodology will provide a transparent assessment of evidence quality across outcomes, enabling clinicians and researchers to understand the certainty of evidence supporting specific OCT biomarkers. Fourth, the assessment of outcomes at multiple time points (3, 6, 12, and 24 months) will enable examination of temporal relationships between baseline OCT biomarkers and visual recovery patterns, as visual improvement following macular hole surgery can continue for up to 24 months postoperatively, with different OCT biomarkers potentially showing varying predictive accuracy at different time points [29]. Lastly, the planned subgroup analyses based on clinically relevant factors such as hole size, surgical technique, and risk of bias will provide additional insights into the contextual applicability of OCT biomarkers. These analyses will help identify whether certain biomarkers perform better in specific clinical scenarios, potentially enabling more personalized prognostic counseling.

Some limitations are anticipated. Methodological heterogeneity in OCT biomarker definitions, measurement protocols, and imaging platforms across studies may not allow us to perform a meta-analysis for certain parameters, particularly newer biomarkers where standardized measurement techniques are not yet established. Variability in surgical techniques, particularly regarding ILM manipulation strategies, may introduce additional heterogeneity that could impact the biomarker-outcome relationships. While we plan to address this through subgroup analysis, the complex interplay between OCT findings and surgical approach may limit our ability to isolate the independent predictive value of specific biomarkers. Moreover, the number of studies contributing to individual subgroups may be limited, reducing the reliability of subgroup findings; we will therefore restrict subgroup analyses to strata with at least two studies, interpret subgroup effects cautiously, and formally assess their credibility using the ICEMAN tool. Finally, postoperative factors such as cataract progression in phakic eyes and the presence of silicone oil tamponade at the time of visual acuity assessment may influence measured visual outcomes; we will record these factors during data extraction and explore their impact in sensitivity analyses.

The findings from this systematic review may have important implications for clinical practice implementation. Evidence synthesis may guide patient counseling regarding realistic visual expectations based on preoperative OCT findings. Furthermore, identification of robust prognostic biomarkers could support risk stratification for surgical technique selection, potentially optimizing outcomes for patients with specific OCT profiles. The review findings may also inform clinical guideline development for macular hole management, providing evidence-based recommendations for prognostic assessment and standardizing the use of OCT biomarkers in clinical practice. This standardization could improve consistency in patient counseling across different centers and practitioners.

### 3.1. Conclusions

This systematic review represents a critical step toward evidence-based implementation of OCT biomarkers in macular hole management. By synthesizing current evidence on the predictive value of baseline OCT biomarkers, this study will clarify their clinical utility and may inform individualized prognostic counseling and surgical decision-making. The rigorous methodology and comprehensive approach employed in this review will provide clinicians and researchers with high-quality evidence to guide both clinical practice and future research directions.

The anticipated findings will contribute to the broader goal of personalized medicine in vitreoretinal surgery, moving beyond the current “one-size-fits-all” approach to more individualized treatment strategies based on objective, quantifiable predictive factors.

## Supporting information

S1 File PRISMA-P checklist. (DOCX)
