# Supplementary material for: OphthoEvidence report: Baseline structural optical coherence tomography biomarkers as predictors of visual outcomes following full-thickness macular hole surgery: A systematic review and meta-analysis protocol
Source: PLoS One. 2026 Sep 30;21(9):e0359108. doi: 10.1371/journal.pone.0359108 (PMC13626361; doi:10.1371/journal.pone.0359108)
Supplement: Supplementary file 2 [file pone.0359108.s003.docx]

**Response to Reviewers**

**Manuscript ID: PONE-D-25-67912**

*Baseline Structural Optical Coherence Tomography Biomarkers as Predictors of Visual Outcomes Following Full-Thickness Macular Hole Surgery: A Systematic Review and Meta-Analysis Protocol*

Dear Dr. Torrado Pacheco,

We thank the Editor and the Reviewer for the careful and constructive evaluation of our protocol. We are grateful for the opportunity to submit a revised version. We have addressed each point in full; our responses are provided below. We believe these revisions address all reviewer concerns and further strengthen the manuscript.

Sincerely,

Varun Chaudhary, MD, PhD, on behalf of all co-authors

Professor of Surgery, Department of Surgery, McMaster University, Hamilton, ON, Canada

**Journal Requirements**

**Formatting and style:**

The manuscript and files have been reformatted to meet PLOS ONE style and file-	naming requirements.

**Funding and Competing Interests:**

We have updated the Funding Statement and Competing Interests Statement, including	the commercial affiliations and funders, together with the required adherence statement;	these have been entered through the online submission system.

# **Response to Reviewer #1**

Reviewer #1: “*The authors present the study protocol for a proposed meta-analysis about the relationship between preoperative OCT biomarkers and visual recovery after macular hole surgery. I feel the inclusion, exclusion criteria should be robust. The authors have not mentioned about the lens status if combined phacoemulsification is not done, axial length, myopic refractive power, presence of retinal detachment or foveoschisis, previous history of trauma, macular edema, etc. Postoperative OCT features have not been mentioned for analysis*.”

**Response**: We thank Reviewer #1 for these constructive comments. We have revised the inclusion and exclusion criteria to be clearer and more robust (Table 1, p. 5–6) and address each point below:

- **Lens status and combined phacoemulsification:** In our eligibility criteria we noted that procedures performed as PPV alone or as combined phacovitrectomy are eligible (Section 2.2, p. 4, line 89). We have now made explicit that the review does not restrict by baseline lens status, and that both phakic and pseudophakic eyes are eligible (Section 2.2, p. 4, line 89; Table 1, p. 5). We have also added lens management as a planned subgroup (PPV alone versus combined phacovitrectomy) (Section 2.7, p. 9, line 163).
- **Axial length and myopic refractive power:** We have clarified in our inclusion criteria that myopic macular holes are eligible (Table 1, p. 5). We have added axial length and refractive error (spherical equivalent) to data extraction (Table 2, p. 7), and we have added a planned subgroup of myopia versus non-myopia) (Section 2.7, p. 9, lines 163–164).
- **Presence of retinal detachment or foveoschisis:** We have added these to our exclusion criteria, as macular hole associated with retinal detachment (MHRD) or with myopic traction maculopathy / foveoschisis will be excluded (Table 1, p. 6).
- **Previous history of trauma:** Traumatic macular holes will be excluded and we have made it clear in our eligibility criteria (Table 1, p. 6).
- **Macular oedema and other co-pathology:** Holes secondary to oedema-producing disease are excluded under our secondary-hole criterion (e.g., associated with diabetic retinopathy, retinal vein occlusion, or other maculopathy) (Table 1, p. 6). For included eyes, relevant ocular comorbidity will be recorded at extraction (Table 2, p. 7).
- **Postoperative OCT features:** This is a deliberate design choice rather than an omission. The review question concerns preoperative (baseline) OCT biomarkers, with the objective of identifying, patients at risk of poorer visual outcomes and supporting preoperative counselling. The predictive value of postoperative OCT recovery markers is a related but separate question, which we identify as a direction for future work. We have made this clear in our eligibility criteria (p. 5).
