# Supplementary material for: OphthoEvidence report: Baseline structural optical coherence tomography biomarkers as predictors of visual outcomes following full-thickness macular hole surgery: A systematic review and meta-analysis protocol
Source: PLoS One. 2026 Sep 30;21(9):e0359108. doi: 10.1371/journal.pone.0359108 (PMC13626361; doi:10.1371/journal.pone.0359108)
Supplement: Supplementary file 3 [file pone.0359108.s004.docx]

**Response to Reviewers**

**Manuscript ID: PONE-D-25-67912**

*Baseline Structural Optical Coherence Tomography Biomarkers as Predictors of Visual Outcomes Following Full-Thickness Macular Hole Surgery: A Systematic Review and Meta-Analysis Protocol*

*Date: 17/Aug/2026*

*Second revision*

Dear Dr. Alvaro Jose Mejia-Vergara,

We thank the Editor and the Reviewer for the careful and constructive evaluation of our protocol. We are grateful for the opportunity to submit a second revised version. We have addressed each point in full; our responses are provided below. We believe these revisions address all reviewer concerns and further strengthen the manuscript.

Sincerely,

Varun Chaudhary, MD, PhD, on behalf of all co-authors

Professor of Surgery, Department of Surgery, McMaster University, Hamilton, ON, Canada

# **Response to Reviewer #2**

**Comment 1: “***The authors propose a study protocol for meta-analysis to examine pre-operative OCT biomarkers’ impact on visual recovery after macular hole surgery. Compared to the predominant existing literature, the authors are taking a very encompassing approach, including studies irrespective of surgical technique (flaps, plugs, tamponade, positioning etc.) While this will allow increased studies, it certainly could confound the conclusions, as the authors note in their limitations. While they hope to address this through subgroup analysis, the number of studies in each subgroup may be too limited to give reliable conclusions given the complexity of findings.*”

**Response**: We agree that variability in surgical technique is a potential source of heterogeneity, and we appreciate the reviewer highlighting the risk that individual subgroups may contain too few studies to support reliable conclusions. Our rationale for the encompassing approach is that this is a prognostic factor review rather than an intervention review: the research question concerns the association between baseline OCT biomarkers and postoperative outcomes, not the comparative effectiveness of surgical techniques. Although the benefits and harms of surgery may vary by surgical technique, the prognostic value of OCT biomarkers is expected to remain relatively consistent across techniques. This is reinforced by the fact that surgical practice for full-thickness macular hole is not standardized. Restricting eligibility to a single technique would answer a narrower question with reduced generalizability, it would also exclude a substantial proportion of the available evidence.

We have implemented several safeguards against over-interpretation. First, meta-analysis will only be performed when two or more studies demonstrate sufficient homogeneity in population characteristics, OCT measurement methods, outcome measures, and statistical reporting (P9, Section 2.6); otherwise findings will be synthesized narratively following SWiM guidance. Second, we have now prespecified *surgical technique* as a confounder that studies should adjust for; otherwise, we will rate the QUIPS confounding domain as being at high risk of bias (P11. Section 2.8)

This will subsequently be reflected in our GRADE certainty ratings (P11, Section 2.8). Third, in response to this comment, we have now specified in Section 2.7 that subgroup analyses will be performed only when at least two studies are available within each subgroup, and that subgroup findings will be interpreted cautiously given potentially limited study numbers; credibility of any apparent subgroup effect will be formally assessed with the ICEMAN tool, which guards against over-interpretation of spurious subgroup differences (P10, Section 2.7). We have also expanded the limitations paragraph in the Discussion to acknowledge this issue explicitly:

“Moreover, the number of studies contributing to individual subgroups may be limited, reducing the reliability of subgroup findings; we will therefore restrict subgroup analyses to strata with at least two studies, interpret subgroup effects cautiously, and formally assess their credibility using the ICEMAN tool. Finally, postoperative factors such as cataract progression in phakic eyes and the presence of silicone oil tamponade at the time of visual acuity assessment may influence measured visual outcomes; we will record these factors during data extraction and explore their impact in sensitivity analyses. (P13. Discussion)”

Changes: Section 2.7 (new sentence on minimum studies per subgroup and cautious	interpretation); Section 2.8 (new variable for assessing the risk of bias – surgical	technique)Discussion, limitations paragraph (new sentences on limited subgroup study	numbers).

**Comment 2:** *“Relatedly, the protocol should be defined and consistent throughout, for example, the initial description includes graft/plugs for example, but Table 1 does not.”*

**Response**: We thank the reviewer for identifying this discrepancy. Table 1 has been revised to mirror Section 2.2: the Population inclusion criteria now explicitly list primary graft/plug techniques (autologous neurosensory retinal transplant, autologous lens capsule flap, human amniotic membrane plug, and autologous platelet/serum adjuncts). We have also reviewed the full eligibility text of Section 2.2 and Table 1 for consistency.

Changes: Table 1, Population inclusion row (new bullet on graft/plug techniques);

**Comment 3:** *“As visual acuity is the main outcome measure, post-operative phakic status and oil presence should also be clearly denoted, as these will potential impact the visual acuity.”*

**Response**: We agree that visual acuity measured with silicone oil in situ, or in eyes with progressing cataract, is not directly comparable to visual acuity measured after oil removal or cataract extraction. We have made four revisions in response. First, the data extraction framework (Table 2) now includes tamponade status at the time of outcome assessment (silicone oil in situ versus removed) and lens status at outcome assessment, including cataract surgery performed during follow-up. Second, Section 2.6 now prespecifies sensitivity analyses excluding studies in which visual acuity was assessed with silicone oil in situ. Third, Section 2.8 now specifies that the QUIPS outcome measurement domain will consider whether visual acuity was assessed under silicone oil or in eyes with visually significant cataract, as these factors may systematically bias measured acuity. Forth, in Section 2.6, we also have noted that when available, we will extract stratified results based on lens and silicone oil status as they may affect BCVA outcome measures. We note that our eligibility criteria and planned subgroup analyses already distinguish PPV alone from combined phacovitrectomy, which partly addresses lens status at the design level.

Changes: Table 2, Surgical interventions row (new extraction elements); Section 2.6	(new sensitivity analysis); Section 2.8 (new outcome measurement consideration);	Discussion, limitations paragraph.

**Comment 4:** *“In general, the authors will have to be very thorough in their analysis to give confidence that their studies are homogenous enough for the meta-analysis to be valid. Alternatively, another approach would be to further define and limit the scope of inclusion criteria.”*

**Response**: We have elected to retain the prespecified scope and to address heterogeneity through rigorous analysis, for the reasons outlined in our response to Comment 1: narrowing eligibility would reduce the clinical generalizability of a prognostic question that is inherently asked across surgical approaches. Our protocol contains an explicit homogeneity safegaurd before any pooling (Section 2.6): studies will only be combined when they are sufficiently similar in population characteristics, OCT biomarker definitions and measurement methods, outcome measures, and statistical reporting; where this is not met, we will synthesize narratively under SWiM rather than force a pooled estimate. Heterogeneity of pooled estimates will be examined through visual inspection of forest plots, statistical tests, and the I-squared statistic, and inconsistency will directly informGRADE certainty ratings. Together with the QUIPS confounding assessment, the newly specified minimum of two studies per subgroup, ICEMAN credibility assessment, and the new sensitivity analyses regarding silicone oil, we believe these measures provide the thoroughness the reviewer requests while preserving the breadth needed to answer the prognostic question comprehensively.

Changes: Sections 2.6, 2.7, 2.8, Table 2, and the Discussion limitations paragraph, as detailed above.
