# Supplementary material for: OphthoEvidence report: Baseline structural optical coherence tomography biomarkers as predictors of visual outcomes following full-thickness macular hole surgery: A systematic review and meta-analysis protocol
Source: PLoS One. 2026 Sep 30;21(9):e0359108. doi: 10.1371/journal.pone.0359108 (PMC13626361; doi:10.1371/journal.pone.0359108)
Supplement: Peer review history

# Peer review: Decision Letter 0

**DOI:** 10.1371/journal.pone.0359108.r001

**Authors:** Alejandro Torrado Pacheco

11 May 2026

Dear Dr. Chaudhary,

Thank you for submitting your manuscript to PLOS ONE. After careful consideration, we feel that it has merit but does not fully meet PLOS ONE’s publication criteria as it currently stands. Therefore, we invite you to submit a revised version of the manuscript that addresses the points raised during the review process.

The reviewer's comments are available below. Could you please address all comments carefully?

Please submit your revised manuscript by Jun 25 2026 11:59PM. If you will need more time than this to complete your revisions, please reply to this message or contact the journal office at plosone@plos.org. When you're ready to submit your revision, log on to https://www.editorialmanager.com/pone/ and select the 'Submissions Needing Revision' folder to locate your manuscript file.

- A letter that responds to each point raised by the academic editor and reviewer(s). You should upload this letter as a separate file labeled 'Response to Reviewers'.
- A marked-up copy of your manuscript that highlights changes made to the original version. You should upload this as a separate file labeled 'Revised Manuscript with Track Changes'.
- An unmarked version of your revised paper without tracked changes. You should upload this as a separate file labeled 'Manuscript'.

If you would like to make changes to your financial disclosure, please include your updated statement in your cover letter. Guidelines for resubmitting your figure files are available below the reviewer comments at the end of this letter.

If applicable, we recommend that you deposit your laboratory protocols in protocols.io to enhance the reproducibility of your results. Protocols.io assigns your protocol its own identifier (DOI) so that it can be cited independently in the future. For instructions see: https://journals.plos.org/plosone/s/submission-guidelines#loc-laboratory-protocols. Additionally, PLOS ONE offers an option for publishing peer-reviewed Lab Protocol articles, which describe protocols hosted on protocols.io. Read more information on sharing protocols at https://plos.org/protocols?utm_medium=editorial-email&utm_source=authorletters&utm_campaign=protocols.

We look forward to receiving your revised manuscript.

Kind regards,

Alejandro Torrado Pacheco, PhD

Associate Editor

PLOS One

Journal Requirements:

When submitting your revision, we need you to address these additional requirements.

1. Please ensure that your manuscript meets PLOS ONE's style requirements, including those for file naming. The PLOS ONE style templates can be found at https://journals.plos.org/plosone/s/file?id=wjVg/PLOSOne_formatting_sample_main_body.pdf and https://journals.plos.org/plosone/s/file?id=ba62/PLOSOne_formatting_sample_title_authors_affiliations.pdf

2. Thank you for stating the following in the Competing Interests section:

[I have read the journal's policy and the authors of this manuscript have the following competing interests: SK: None. JPL: None. DZ: None. SM: None. SK: None. CCW: CCW reports consulting for 4DMT, AbbVie, Adverum, Alcon, Alimera, Alkeus, Annexon, Apellis, Aviceda, Bayer, Biocryst, Boehringer Ingelheim, Clearside, EyeBiotech, EyePoint, Genentech, InGel, Janssen, Kiora, KodiakMerck, Neurotech, Novartis, Ocuphire, ONL, Opthea, Osanni, Panther, Perceive Bio, Ray, Regeneron, RegenXBio, Sanofi, Santen, Stealth, Valo, Zeiss. PKK: reports consulting for Genentech, Alcon, Allergan, and Novartis and having received lecture fees from Novartis and Genentech. TYW: TYW is a consultant for Astellas, Bayer, Boehringer-Ingelheim, Genentech, Iveric Bio, Novartis, Oxurion, Plano, Roche, Sanofi, and Shanghai Henlius. He is an inventor, holds patents and is a co-founder of start-up companies EyRiS and Visre, which have interests in and develop digital solutions for eye diseases. JC: None. ML: None. VC: VC reports acting as an advisory board member, grants and other from Novartis; acting as an advisory board member, grants and other from Bayer; grants from Allergan; acting as an advisory board member and grants from Roche; acting as an advisory board member for Janssen; acting as an advisory board member for Apellis; acting as an advisory board member for Boehringer Ingelheim, and acting as an advisory board member for EyePoint outside the submitted work.].

We note that one or more of the authors are employed by commercial companies: EyRiS, Visre, Retina Consultants of Texas.

1. Please provide an amended Funding Statement declaring this commercial affiliation, as well as a statement regarding the Role of Funders in your study. If the funding organization did not play a role in the study design, data collection and analysis, decision to publish, or preparation of the manuscript and only provided financial support in the form of authors' salaries and/or research materials, please review your statements relating to the author contributions, and ensure you have specifically and accurately indicated the role(s) that these authors had in your study. You can update author roles in the Author Contributions section of the online submission form.

Please also include the following statement within your amended Funding Statement.

“The funder provided support in the form of salaries for authors [insert relevant initials], but did not have any additional role in the study design, data collection and analysis, decision to publish, or preparation of the manuscript. The specific roles of these authors are articulated in the ‘author contributions’ section.”

If your commercial affiliation did play a role in your study, please state and explain this role within your updated Funding Statement.

2. Please also provide an updated Competing Interests Statement declaring this commercial affiliation along with any other relevant declarations relating to employment, consultancy, patents, products in development, or marketed products, etc.

Within your Competing Interests Statement, please confirm that this commercial affiliation does not alter your adherence to all PLOS ONE policies on sharing data and materials by including the following statement: "This does not alter our adherence to PLOS ONE policies on sharing data and materials.” (as detailed online in our guide for authors http://journals.plos.org/plosone/s/competing-interests) . If this adherence statement is not accurate and there are restrictions on sharing of data and/or materials, please state these. Please note that we cannot proceed with consideration of your article until this information has been declared.

Please include both an updated Funding Statement and Competing Interests Statement in your cover letter. We will change the online submission form on your behalf.

3. Thank you for stating the following in the Competing Interests section:

[I have read the journal's policy and the authors of this manuscript have the following competing interests: SK: None. JPL: None. DZ: None. SM: None. SK: None. CCW: CCW reports consulting for 4DMT, AbbVie, Adverum, Alcon, Alimera, Alkeus, Annexon, Apellis, Aviceda, Bayer, Biocryst, Boehringer Ingelheim, Clearside, EyeBiotech, EyePoint, Genentech, InGel, Janssen, Kiora, KodiakMerck, Neurotech, Novartis, Ocuphire, ONL, Opthea, Osanni, Panther, Perceive Bio, Ray, Regeneron, RegenXBio, Sanofi, Santen, Stealth, Valo, Zeiss. PKK: reports consulting for Genentech, Alcon, Allergan, and Novartis and having received lecture fees from Novartis and Genentech. TYW: TYW is a consultant for Astellas, Bayer, Boehringer-Ingelheim, Genentech, Iveric Bio, Novartis, Oxurion, Plano, Roche, Sanofi, and Shanghai Henlius. He is an inventor, holds patents and is a co-founder of start-up companies EyRiS and Visre, which have interests in and develop digital solutions for eye diseases. JC: None. ML: None. VC: VC reports acting as an advisory board member, grants and other from Novartis; acting as an advisory board member, grants and other from Bayer; grants from Allergan; acting as an advisory board member and grants from Roche; acting as an advisory board member for Janssen; acting as an advisory board member for Apellis; acting as an advisory board member for Boehringer Ingelheim, and acting as an advisory board member for EyePoint outside the submitted work.].

We note that you received funding from commercial sources: Bayer, Novartis, Roche, Genentech, Allergan, EyePoint Pharmaceuticals, AbbVie, Astellas, Boehringer Ingelheim, Janssen, Merck, Sanofi, Santen, 4DMT, Adverum, Alkeus, Annexon, Apellis, Aviceda, Biocryst, Clearside, EyeBiotech, EyePoint, Iveric Bio, Kodiak, Neurotech, Opthea, Oxurion, Ray, RegenXBio, Stealth, Zeiss, Alcon, Shanghai Henlius, Ocuphire, ONL, Panther, Perceive Bio, and Valo.

Please provide an amended Competing Interests Statement that explicitly states these commercial funders, along with any other relevant declarations relating to employment, consultancy, patents, products in development, marketed products, etc.

Within this Competing Interests Statement, please confirm that this does not alter your adherence to all PLOS ONE policies on sharing data and materials by including the following statement: "This does not alter our adherence to PLOS ONE policies on sharing data and materials.” (as detailed online in our guide for authors http://journals.plos.org/plosone/s/competing-interests).  If there are restrictions on sharing of data and/or materials, please state these. Please note that we cannot proceed with consideration of your article until this information has been declared.

Please include your amended Competing Interests Statement within your cover letter. We will change the online submission form on your behalf.

If the reviewer comments include a recommendation to cite specific previously published works, please review and evaluate these publications to determine whether they are relevant and should be cited. There is no requirement to cite these works unless the editor has indicated otherwise.

[Note: HTML markup is below. Please do not edit.]

Reviewers' comments:

Reviewer's Responses to Questions

**Comments to the Author**

1. Does the manuscript provide a valid rationale for the proposed study, with clearly identified and justified research questions?

Reviewer #1: Yes

**********

2. Is the protocol technically sound and planned in a manner that will lead to a meaningful outcome and allow testing the stated hypotheses?

Reviewer #1: Partly

**********

3. Is the methodology feasible and described in sufficient detail to allow the work to be replicable?

Reviewer #1: Yes

**********

4. Have the authors described where all data underlying the findings will be made available when the study is complete?

The PLOS Data policy

Reviewer #1: No

**********

5. Is the manuscript presented in an intelligible fashion and written in standard English?

Reviewer #1: Yes

**********

Please use the space provided to explain your answers to the questions above and, if applicable, provide comments about issues authors must address before this protocol can be accepted for publication. You may also include additional comments for the author, including concerns about research or publication ethics.

You may also provide optional suggestions and comments to authors that they might find helpful in planning their study.

Reviewer #1: The authors present the study protocol for a proposed meta analysis about the relationship between preoperative OCT biomarkers and visual recovery after macular hole surgery. I feel the inclusion, exclusion criteria should be robust. The authors have not mentioned about the lens status if combined phacoemulsification is not done, axial length, myopic refractive power, presence of retinal detachment or foveoschisis, previous history of trauma, macular edema, etc. Postoperative OCT features have not been mentioned for analysis.

**********

what does this mean?). If published, this will include your full peer review and any attached files.

If you choose “no”, your identity will remain anonymous but your review may still be made public.

**Do you want your identity to be public for this peer review?**  For information about this choice, including consent withdrawal, please see our Privacy Policy

Reviewer #1: No

**********

[NOTE: If reviewer comments were submitted as an attachment file, they will be attached to this email and accessible via the submission site. Please log into your account, locate the manuscript record, and check for the action link "View Attachments". If this link does not appear, there are no attachment files.]

To ensure your figures meet our technical requirements, please review our figure guidelines: https://journals.plos.org/plosone/s/figures

You may also use PLOS’s free figure tool, NAAS, to help you prepare publication quality figures: https://journals.plos.org/plosone/s/figures#loc-tools-for-figure-preparation.

NAAS will assess whether your figures meet our technical requirements by comparing each figure against our figure specifications.

# Author comment: Author response to Decision Letter 1

**DOI:** 10.1371/journal.pone.0359108.r002

9 Jun 2026

Response to Reviewers

Manuscript ID: PONE-D-25-67912

Baseline Structural Optical Coherence Tomography Biomarkers as Predictors of Visual Outcomes Following Full-Thickness Macular Hole Surgery: A Systematic Review and Meta-Analysis Protocol

Dear Dr. Torrado Pacheco,

We thank the Editor and the Reviewer for the careful and constructive evaluation of our protocol. We are grateful for the opportunity to submit a revised version. We have addressed each point in full; our responses are provided below. We believe these revisions address all reviewer concerns and further strengthen the manuscript.

Sincerely,

Varun Chaudhary, MD, PhD, on behalf of all co-authors

Professor of Surgery, Department of Surgery, McMaster University, Hamilton, ON, Canada

Journal Requirements

Formatting and style:

The manuscript and files have been reformatted to meet PLOS ONE style and file- naming requirements.

Funding and Competing Interests:

We have updated the Funding Statement and Competing Interests Statement, including the commercial affiliations and funders, together with the required adherence statement; these have been entered through the online submission system.

Response to Reviewer #1

Reviewer #1: “The authors present the study protocol for a proposed meta-analysis about the relationship between preoperative OCT biomarkers and visual recovery after macular hole surgery. I feel the inclusion, exclusion criteria should be robust. The authors have not mentioned about the lens status if combined phacoemulsification is not done, axial length, myopic refractive power, presence of retinal detachment or foveoschisis, previous history of trauma, macular edema, etc. Postoperative OCT features have not been mentioned for analysis.”

Response: We thank Reviewer #1 for these constructive comments. We have revised the inclusion and exclusion criteria to be clearer and more robust (Table 1, p. 5–6) and address each point below:

• Lens status and combined phacoemulsification: In our eligibility criteria we noted that procedures performed as PPV alone or as combined phacovitrectomy are eligible (Section 2.2, p. 4, line 89). We have now made explicit that the review does not restrict by baseline lens status, and that both phakic and pseudophakic eyes are eligible (Section 2.2, p. 4, line 89; Table 1, p. 5). We have also added lens management as a planned subgroup (PPV alone versus combined phacovitrectomy) (Section 2.7, p. 9, line 163).

• Axial length and myopic refractive power: We have clarified in our inclusion criteria that myopic macular holes are eligible (Table 1, p. 5). We have added axial length and refractive error (spherical equivalent) to data extraction (Table 2, p. 7), and we have added a planned subgroup of myopia versus non-myopia) (Section 2.7, p. 9, lines 163–164).

• Presence of retinal detachment or foveoschisis: We have added these to our exclusion criteria, as macular hole associated with retinal detachment (MHRD) or with myopic traction maculopathy / foveoschisis will be excluded (Table 1, p. 6).

• Previous history of trauma: Traumatic macular holes will be excluded and we have made it clear in our eligibility criteria (Table 1, p. 6).

• Macular oedema and other co-pathology: Holes secondary to oedema-producing disease are excluded under our secondary-hole criterion (e.g., associated with diabetic retinopathy, retinal vein occlusion, or other maculopathy) (Table 1, p. 6). For included eyes, relevant ocular comorbidity will be recorded at extraction (Table 2, p. 7).

• Postoperative OCT features: This is a deliberate design choice rather than an omission. The review question concerns preoperative (baseline) OCT biomarkers, with the objective of identifying, patients at risk of poorer visual outcomes and supporting preoperative counselling. The predictive value of postoperative OCT recovery markers is a related but separate question, which we identify as a direction for future work. We have made this clear in our eligibility criteria (p. 5).

Attachment Submitted filename: Response_to_Reviewers_PONE-D-25-67912.docx

# Peer review: Decision Letter 1

**DOI:** 10.1371/journal.pone.0359108.r003

**Authors:** Alvaro Mejia-Vergara

12 Aug 2026

Dear Dr. Chaudhary,

Thank you for submitting your manuscript to PLOS ONE. After careful consideration, we feel that it has merit but does not fully meet PLOS ONE’s publication criteria as it currently stands. Therefore, we invite you to submit a revised version of the manuscript that addresses the points raised during the review process.

The main concern for the reviewers and myself is the level of analysis that is required to keep all the sub announces ballot with this very large endeavor. We want you to provide a more explanation how you are going to manage all this annelids.

Please submit your revised manuscript by Sep 26 2026 11:59PM. If you will need more time than this to complete your revisions, please reply to this message or contact the journal office at plosone@plos.org. When you're ready to submit your revision, log on to https://www.editorialmanager.com/pone/ and select the 'Submissions Needing Revision' folder to locate your manuscript file.

- A letter that responds to each point raised by the academic editor and reviewer(s). You should upload this letter as a separate file labeled 'Response to Reviewers'.
- A marked-up copy of your manuscript that highlights changes made to the original version. You should upload this as a separate file labeled 'Revised Manuscript with Track Changes'.
- An unmarked version of your revised paper without tracked changes. You should upload this as a separate file labeled 'Manuscript'.

If you would like to make changes to your financial disclosure, please include your updated statement in your cover letter. Guidelines for resubmitting your figure files are available below the reviewer comments at the end of this letter.

If applicable, we recommend that you deposit your laboratory protocols in protocols.io to enhance the reproducibility of your results. Protocols.io assigns your protocol its own identifier (DOI) so that it can be cited independently in the future. For instructions see: https://journals.plos.org/plosone/s/submission-guidelines#loc-laboratory-protocols. Additionally, PLOS ONE offers an option for publishing peer-reviewed Lab Protocol articles, which describe protocols hosted on protocols.io. Read more information on sharing protocols at https://plos.org/protocols?utm_medium=editorial-email&utm_source=authorletters&utm_campaign=protocols.

As the corresponding author, your ORCID iD is verified in the submission system and will appear in the published article. PLOS supports the use of ORCID, and we encourage all coauthors to register for an ORCID iD and use it as well. Please encourage your coauthors to verify their ORCID iD within the submission system before final acceptance, as unverified ORCID iDs will not appear in the published article. *Only*  the individual author can complete the verification step; PLOS staff *cannot*  verify ORCID iDs on behalf of authors.

We look forward to receiving your revised manuscript.

Kind regards,

Alvaro Jose Mejia-Vergara, MD

Academic Editor

PLOS One

Journal Requirements:

If the reviewer comments include a recommendation to cite specific previously published works, please review and evaluate these publications to determine whether they are relevant and should be cited. There is no requirement to cite these works unless the editor has indicated otherwise.

Please review your reference list to ensure that it is complete and correct. If you have cited papers that have been retracted, please include the rationale for doing so in the manuscript text, or remove these references and replace them with relevant current references. Any changes to the reference list should be mentioned in the rebuttal letter that accompanies your revised manuscript. If you need to cite a retracted article, indicate the article’s retracted status in the References list and also include a citation and full reference for the retraction notice.

[Note: HTML markup is below. Please do not edit.]

Reviewers' comments:

Reviewer's Responses to Questions

**Comments to the Author**

1. Does the manuscript provide a valid rationale for the proposed study, with clearly identified and justified research questions?

Reviewer #2: Yes

**********

2. Is the protocol technically sound and planned in a manner that will lead to a meaningful outcome and allow testing the stated hypotheses?

Reviewer #2: Partly

**********

3. Is the methodology feasible and described in sufficient detail to allow the work to be replicable?

Reviewer #2: Yes

**********

4. Have the authors described where all data underlying the findings will be made available when the study is complete?

The PLOS Data policy

Reviewer #2: Yes

**********

5. Is the manuscript presented in an intelligible fashion and written in standard English?

Reviewer #2: Yes

**********

Please use the space provided to explain your answers to the questions above and, if applicable, provide comments about issues authors must address before this protocol can be accepted for publication. You may also include additional comments for the author, including concerns about research or publication ethics.

You may also provide optional suggestions and comments to authors that they might find helpful in planning their study.

Reviewer #2: The authors propose a study protocol for meta-analysis to examine pre-operative OCT biomarkers’ impact on visual recovery after macular hole surgery. Compared to the predominant existing literature, the authors are taking a very encompassing approach, including studies irrespective of surgical technique (flaps, plugs, tamponade, positioning etc.) While this will allow increased studies, it certainly could confound the conclusions, as the authors note in their limitations. While they hope to address this through subgroup analysis, the number of studies in each subgroup may be too limited to give reliable conclusions given the complexity of findings. Relatedly, the protocol should be defined and consistent throughout, for example, the initial description includes graft/plugs for example, but Table 1 does not.

As visual acuity is the main outcome measure, post-operative phakic status and oil presence should also be clearly denoted, as these will potential impact the visual acuity. In genearl, the authors will have to be very thorough in their analysis to give confidence that their studies are homogenous enough for the meta-analysis to be valid. Alternatively, another approach would be to further define and limit the scope of inclusion criteria.

**********

what does this mean?). If published, this will include your full peer review and any attached files.

If you choose “no”, your identity will remain anonymous but your review may still be made public.

**Do you want your identity to be public for this peer review?**  For information about this choice, including consent withdrawal, please see our Privacy Policy

Reviewer #2: No

**********

[NOTE: If reviewer comments were submitted as an attachment file, they will be attached to this email and accessible via the submission site. Please log into your account, locate the manuscript record, and check for the action link "View Attachments". If this link does not appear, there are no attachment files.]

To ensure your figures meet our technical requirements, please review our figure guidelines: https://journals.plos.org/plosone/s/figures

You may also use PLOS’s free figure tool, NAAS, to help you prepare publication quality figures: https://journals.plos.org/plosone/s/figures#loc-tools-for-figure-preparation.

NAAS will assess whether your figures meet our technical requirements by comparing each figure against our figure specifications.

# Author comment: Author response to Decision Letter 2

**DOI:** 10.1371/journal.pone.0359108.r004

18 Aug 2026

Dear Dr. Alvaro Jose Mejia-Vergara,

We thank the Editor and the Reviewer for the careful and constructive evaluation of our protocol. We are grateful for the opportunity to submit a second revised version. We have addressed each point in full; our responses are provided in attached document. We believe these revisions address all reviewer concerns and further strengthen the manuscript.

Sincerely,

Varun Chaudhary, MD, PhD, on behalf of all co-authors

Professor of Surgery, Department of Surgery, McMaster University, Hamilton, ON, Canada

Attachment Submitted filename: Response_to_Reviewers_PONE-D-25-67912_auresp_2.docx

# Peer review: Decision Letter 2

**DOI:** 10.1371/journal.pone.0359108.r005

**Authors:** Alvaro Mejia-Vergara

9 Sep 2026

OphthoEvidence Report: Baseline Structural Optical Coherence Tomography Biomarkers as Predictors of Visual Outcomes Following Full-Thickness Macular Hole Surgery: A Systematic Review and Meta-Analysis Protocol

PONE-D-25-67912R2

Dear Dr. Chaudhary,

We’re pleased to inform you that your manuscript has been judged scientifically suitable for publication and will be formally accepted for publication once it meets all outstanding technical requirements.

Within one week, you’ll receive an e-mail detailing the required amendments. When these have been addressed, you’ll receive a formal acceptance letter and your manuscript will be scheduled for publication.

An invoice will be generated when your article is formally accepted. Please note, if your institution has a publishing partnership with PLOS and your article meets the relevant criteria, all or part of your publication costs will be covered. Please make sure your user information is up-to-date by logging into Editorial Manager at Editorial Manager® and clicking the ‘Update My Information' link at the top of the page. For questions related to billing, please contact billing support.

If your institution or institutions have a press office, please notify them about your upcoming paper to help maximize its impact. If they’ll be preparing press materials, please inform our press team as soon as possible -- no later than 48 hours after receiving the formal acceptance. Your manuscript will remain under strict press embargo until 2 pm Eastern Time on the date of publication. For more information, please contact onepress@plos.org.

Kind regards,

Alvaro Jose Mejia-Vergara, MD

Academic Editor

PLOS One

Additional Editor Comments (optional):

Reviewers' comments:

Reviewer's Responses to Questions

**Comments to the Author**

1. Does the manuscript provide a valid rationale for the proposed study, with clearly identified and justified research questions?

Reviewer #2: Yes

**********

2. Is the protocol technically sound and planned in a manner that will lead to a meaningful outcome and allow testing the stated hypotheses?

Reviewer #2: Yes

**********

3. Is the methodology feasible and described in sufficient detail to allow the work to be replicable?

Reviewer #2: Yes

**********

4. Have the authors described where all data underlying the findings will be made available when the study is complete?

The PLOS Data policy

Reviewer #2: Yes

**********

5. Is the manuscript presented in an intelligible fashion and written in standard English?

Reviewer #2: Yes

**********

Please use the space provided to explain your answers to the questions above and, if applicable, provide comments about issues authors must address before this protocol can be accepted for publication. You may also include additional comments for the author, including concerns about research or publication ethics.

You may also provide optional suggestions and comments to authors that they might find helpful in planning their study.

Reviewer #2: The authors propose a study protocol for meta-analysis to examine preoperative OCT biomarkers’ impact on visual recovery after macular hole surgery. The authors have done a good job addressing the previous comments, and with rigorous analysis, should be able to provide interesting clinical insight.

**********

what does this mean?). If published, this will include your full peer review and any attached files.

If you choose “no”, your identity will remain anonymous but your review may still be made public.

**Do you want your identity to be public for this peer review?**  For information about this choice, including consent withdrawal, please see our Privacy Policy

Reviewer #2: **Yes:** Jonathan K Hu

**********

# Editor report: Acceptance letter

**DOI:** 10.1371/journal.pone.0359108.r006

**Authors:** Alvaro Mejia-Vergara

PONE-D-25-67912R2

PLOS One

Dear Dr. Chaudhary,

I'm pleased to inform you that your manuscript has been deemed suitable for publication in PLOS One. Congratulations! Your manuscript is now being handed over to our production team.

At this stage, our production department will prepare your paper for publication. This includes ensuring the following:

* All references, tables, and figures are properly cited

* All relevant supporting information is included in the manuscript submission,

* There are no issues that prevent the paper from being properly typeset

You will receive further instructions from the production team, including instructions on how to review your proof when it is ready. Please keep in mind that we are working through a large volume of accepted articles, so please give us a few days to review your paper and let you know the next and final steps.

Lastly, if your institution or institutions have a press office, please let them know about your upcoming paper now to help maximize its impact. If they'll be preparing press materials, please inform our press team within the next 48 hours. Your manuscript will remain under strict press embargo until 2 pm Eastern Time on the date of publication. For more information, please contact onepress@plos.org.

You will receive an invoice from PLOS for your publication fee after your manuscript has reached the completed accept phase. If you receive an email requesting payment before acceptance or for any other service, this may be a phishing scheme. Learn how to identify phishing emails and protect your accounts at https://explore.plos.org/phishing.

If we can help with anything else, please email us at customercare@plos.org.

Thank you for submitting your work to PLOS One and supporting open access.

Kind regards,

PLOS One Editorial Office Staff

on behalf of

Dr. Alvaro Jose Mejia-Vergara

Academic Editor

PLOS One
